# Macrolide analog F806 suppresses esophageal squamous cell carcinoma (ESCC) by blocking β1 integrin activation

**DOI:** 10.18632/oncotarget.3612

**Published:** 2015-04-03

**Authors:** Li-Yan Li, Hong Jiang, Yang-Min Xie, Lian-Di Liao, Hui-Hui Cao, Xiu-E Xu, Bo Chen, Fa-Min Zeng, Ying-Li Zhang, Ze-Peng Du, Hong Chen, Wei Huang, Wei Jia, Wei Zheng, Jian-Jun Xie, En-Min Li, Li-Yan Xu

**Affiliations:** ^1^ The Key Laboratory of Molecular Biology for High Cancer Incidence Coastal Chaoshan Area, Shantou University Medical College, Shantou, Guangdong, P.R. China; ^2^ Institute of Oncologic Pathology, Shantou University Medical College, Shantou, Guangdong, P.R. China; ^3^ Fujian Key Laboratory of Screening for Novel Microbial Products, Fujian Institute of Microbiology, Fuzhou, Fujian, P.R. China; ^4^ Experimental Animal Center, Shantou University Medical College, Shantou, Guangdong, P.R. China; ^5^ Department of Biochemistry and Molecular Biology, Shantou University Medical College, Shantou, Guangdong, P.R. China

**Keywords:** macrolide analog F806, β1 integrin, cell adhesion, anoikis, esophageal squamous cell carcinoma cells

## Abstract

The paucity of new drugs for the treatment of esophageal squamous cell carcinoma (ESCC) limits the treatment options. This study characterized the therapeutic efficacy and action mechanism of a novel natural macrolide compound F806 in human ESCC xenograft models and cell lines. F806 inhibited growth of ESCC, most importantly, it displayed fewer undesirable side effects on normal tissues in two human ESCC xenograft models. F806 inhibited proliferation of six ESCC cells lines, with the half maximal inhibitory concentration (IC_50_) ranging from 9.31 to 16.43 μM. Furthermore, F806 induced apoptosis of ESCC cells, contributing to its growth-inhibitory effect. Also, F806 inhibited cell adhesion resulting in anoikis. Mechanistic studies revealed that F806 inhibited the activation of β1 integrin in part by binding to a novel site Arg610 of β1 integrin, suppressed focal adhesion formation, decreased cell adhesion to extracellular matrix and eventually triggered apoptosis. We concluded that F806 would potentially be a well-tolerated anticancer drug by targeting β1 integrin, resulting in anoikis in ESCC cells.

## INTRODUCTION

Esophageal squamous cell carcinoma (ESCC), the major histologic form of esophageal cancer, is one of the most frequent fatal malignancies worldwide and the fourth leading cause of cancer-related death in China [1]. Despite aggressive treatment modalities such as surgical resection with extensive lymphadenectomy and surgery combined with chemotherapy and/or radiotherapy, survival of ESCC patients with recurrence or metastasis remains poor [2, 3]. Therefore, novel safe and effective therapeutic strategies such as the utilization of targeted therapy drugs are highly desirable for the patients with ESCC.

Remarkable advances in cancer molecular biology and pathogenesis have recently led to successful development of myriad mechanism-based targeted therapies that substantially improve survival and quality of life for cancer patients [4]. The vanguard of novel anticancer drugs often targets cellular signaling mechanisms, aberrant tumor stroma, and tumor vasculature and microenvironment [5–7]. Intracellular signaling network which are propagated from the cell surface to intracellular processes, comprising extracellular ligands, transmembrane receptors and cytoplasmic secondary messengers, often optimizes tumor growth and metastasis in malignancies, and represents potential selective targets for cancer therapy [8]. Integrins are a family of the heterodimeric cell surface receptors that bind to the cell extracellular matrix (ECM), promote the transmission of multiple signaling pathways, and coordinate extensive broad range of functional activities, such as cell growth, survival, adhesion, invasion and metastasis [9, 10]. Eighteen α-subunits and eight β-subunits assemble into 24 different integrins [11]. Among these integrins, β1 integrin has recently been shown to play a key role in regulating the switch from a dormant state to active proliferation and metastasis, and confer therapeutic resistance in multiple solid cancer models [12–16]. Importantly, it has been reported several possible therapeutic approaches, such as small molecules or inhibitory antibodies, directing against β1 integrin signaling to prevent the recurrence of cancer in experimental models [17–19]. However, β1 integrin as a strategic molecular target of a novel chemotherapeutic agent has not been well investigated in ESCC.

The natural compound F806, also named FW-04-806 is isolated by fermentation of soil *Streptomyces* sp. FIM-04-806, and possesses both bioxazole and macrodiolide chemical structures (Supplementary Figure 1) [20, 21]. Our previous study has been reported that F806 exhibited potent activity against human cancer cells [22]. In the current study, we investigated the anti-cancer effect of F806 in ESCC cells *in vivo* and *in vitro*, and the biological activity of F806 against ESCC cells by blocking β1 integrin activation.

## RESULTS

### F806 inhibits tumor growth in EC109 and KYSE510 tumor xenograft models

**Figure 1:**
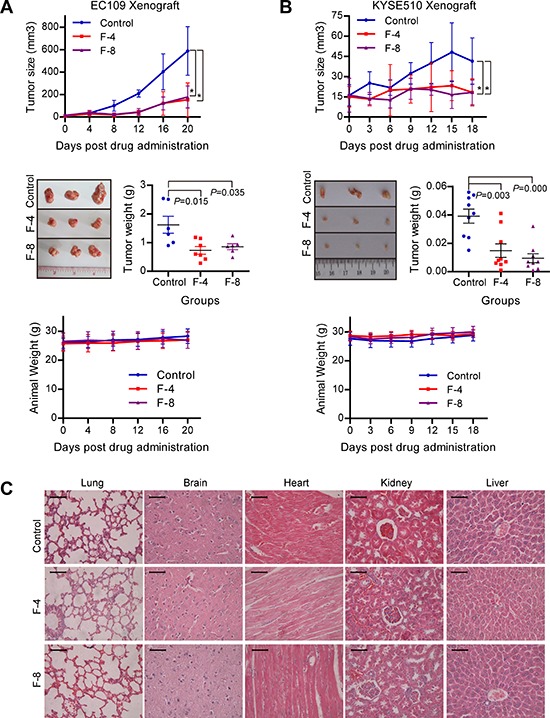
Anti-tumor effect and low toxicity of F806 in ESCC xenograft tumor models **A.** and **B.** F806 inhibited tumor growth of ESCC xenograft models with low toxicity. *Upper panel*: Tumor volume growth curve of xenograft models in control and treated groups. *Middle panel*: Weight and representative pictures of the excised tumors on day 22 in control and treated groups. *Lower panel*: Body weight curve of xenograft mice in treated and control groups. **C.** Representative Hematoxylin-eosin staining for potential toxicity of F806 in lung, brain, heart, kidney and liver of F806-treated mice. Scale bar = 50 μm. *P < 0.05 *vs*. control mice; mean ± SD, *n* = 7; F-4, F806-4 mg/kg; F-8, F806-8 mg/kg.

To assess the antitumor potential of F806 in xenograft models, EC109 and KYSE510 esophageal squamous cell carcinoma (ESCC) cells were inoculated subcutaneously into nude mice. Three groups of xenograft mice were intraperitoneally administered with vehicle (control), 4 mg/kg and 8 mg/kg F806 daily for 21 days respectively and sacrificed at the end. The data showed that tumors from both of F806-treated groups grew more slowly than the control group (Figure 1A and 1B). In detail, no significant difference of the tumor volume in each group was observed at the beginning of treatment. However, a significant (*P* < 0.05) antitumor effect of F806 was displayed in EC109 and KYSE510 xenograft models beginning at day 8/9 after the start of treatment. At the end of treatment, 4 mg/kg or 8 mg/kg F806 reduced tumor growth by 55.0% (*P* = 0.015) or 47.2% (*P* = 0.035) in EC109 cells, and 62.2% (*P* = 0.003) or 75.9% (*P* = 0.000) in KYSE510 cells, as compared to the control group.

Simultaneously, the safety of F806 was evaluated in xenograft mice. All mice tolerated this treatment well without toxic symptoms or signs and had stable body weights during the treatment (Figure 1A and 1B, lower panel). No significance of biochemical markers for liver and renal function was found between F806-treated and control mice (Supplementary Table 3). No effect on complete blood count including white blood, red blood, hemoglobin and blood platelet count, was observed between F806-treated and control mice (Supplementary Table 4). In addition, no histological abnormality was shown in lungs, brains, liver, heart and kidneys of mice between F806-treated and control groups at the end of drug treatment (Figure 1C). Together, these data suggest that F806 effectively inhibits tumor growth in the absence of drug-induced adverse effects.

### F806 inhibits cell proliferation in various ESCC cells

**Figure 2:**
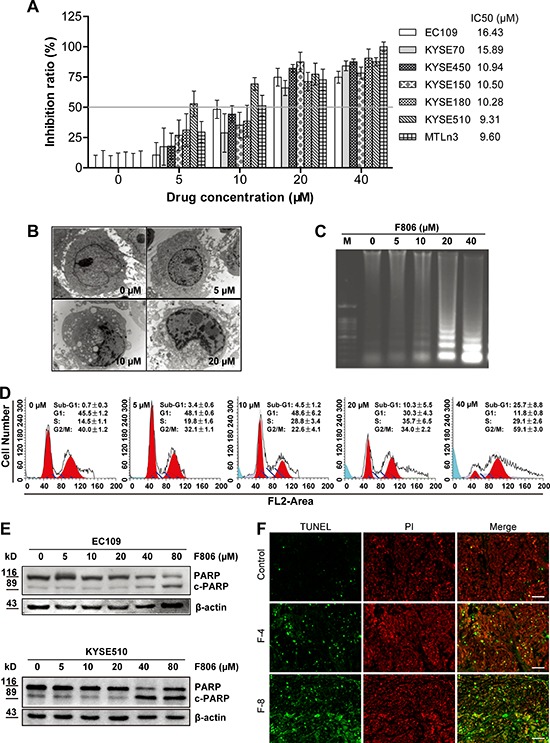
F806 inhibits growth and induces apoptosis in ESCC cells Various ESCC cells were treated with 0 - 40 μM F806 for 24 or 72 hours. **A.** F806 inhibited proliferation of ESCC cells with IC_50_ values ranging from 9.31 to 16.43 μM. Proliferation was measured by MTT assay, and the 72 hr IC_50_ of F806 was evaluated. Mean ± SD; *n* = 12. **B.** Morphological changes of apoptosis were observed by transmission electron microscopy of F806-treated EC109 cells (original magnification, 30,000×). **C.** DNA laddering in F806-treated EC109 cells. **D.** Flow cytometry shows the appearance of a sub-G1 peak in F806-treated EC109 cells. Mean ± SD, *n* = 6. **E.** Western blot analysis for execution of apoptosis in F806-treated ESCC cells. **F.** paraffin-embedded tumor tissues from xenograft models were subjected to DeadEnd Fluorometric TUNEL-assay for detection of apoptosis. The TUNEL-positive cells are visualized in green fluorescence in a red (PI) background by fluorescence microscopy (Original magnification, 400×). F-4, F806-4 mg/kg; F-8, F806-8 mg/kg.

To assess the effects of F806 on cell growth, cell viability was determined by MTT assay in various ESCC cell lines, including EC109, KYSE70, KYSE450, KYSE150, KYSE180, and KYSE510 cells. Meanwhile, as a positive control, the growth of MTLn3 rat mammary adenocarcinoma cell was inhibited by F806 with 72 hr IC_50_ value of 9.60 μM, which is consistent with a previous report [22]. Shown in cell viability assays on ESCC cells, rounding and detachment of cultured cells increased in a dose- (0–40 μM) and time-dependent (0–72 h) manner after treatment with F806 (the morphology features of EC109 cells as shown in Supplementary Figure 2). The growth-inhibitory effect of F806 was tested in various ESCC cell lines at 72 hr, with IC_50_ values of 16.43, 15.89, 10.94, 10.50, 10.28 and 9.31 μM in EC109, KYSE70, KYSE450, KYSE150, KYSE180, and KYSE510 cells respectively (Figure 2A). Notably F806 demonstrated potent growth-inhibitory effects against ESCC cells.

### F806 induces cell apoptosis in ESCC cells

We next examined whether the growth-inhibitory effect of F806 was due to apoptosis. Transmission electron microscopy revealed condensation and margination of nuclear chromatin surrounding in the nucleus of EC109 cells treated with F806 for 24 hr, which is strongly suggestive of apoptotic cell death (Figure 2B). An apoptotic phenotype was further supported by DNA laddering, a specific marker for cell apoptosis (Figure 2C) in F806-treated EC109 cells. On the other hand, cell cycle analysis presented the presence of a sub-G1 DNA peak in F806-treated EC109 cells (Figure 2D). Furthermore, the apoptosis precursor (ADP-ribose) polymerase (PARP) was cleaved, along with the reduction of PARP and the increase of cleaved PARP in F806-treated ESCC cells (Figure 2E). To further confirm the F806-induced apoptosis in ESCC cells, tumor sections from xenograft models were stained for nuclear apoptosis using the DeadEnd Fluorometric TUNEL reagent staining (Figure 2F). In contrast to control group, tumors from the F806-treated group showed obvious nuclear condensation and DNA fragment staining. Thus, F806 would induce apoptosis, contributing to growth inhibition and cell death in ESCC cells.

### F806 inhibits cell adhesion and sensitizes ESCC cells to anoikis

**Figure 3:**
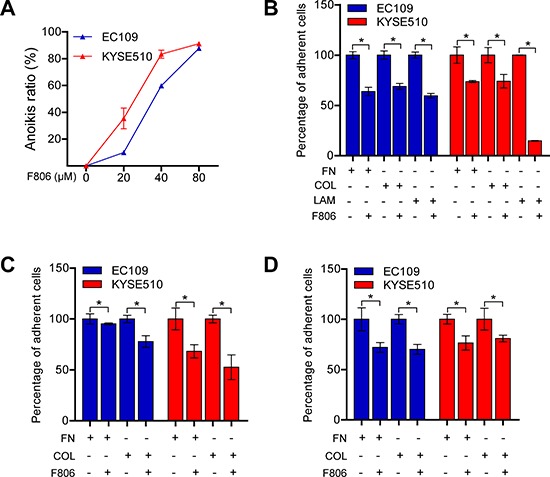
F806 inhibits cell adhesions and induces anoikis in ESCC cells **A.** EC109 and KYSE510 cells were suspension cultured on poly-HEMA-coated plates in the presence of F806 for 48 h and anoikis ratio was then determined by MTT assay. **B.** EC109 and KYSE510 cells were kept in fibronectin (FN), collagen (COL) or laminin (LAM)-coated plates in the presence of F806 for 24 h. **C.** EC109 and KYSE510 cells were kept in FN or COL-coated plates for 30 min and treated with F806 for another 24 h. **D.** EC109 and KYSE510 cells were kept in FN or COL-coated plates for 24 h prior to 30 min of F806 stimulation. The attached cells were recorded by MTT assay in each group. Data represent three independent experiments; *P < 0.05 *vs*. control group; mean ± SD, *n* = 3–6.

ESCC cells, as epithelial cells, require attachment to an appropriate extracellular matrix (ECM) in order to survive [23]. Interestingly, we noted that ESCC cells began to round up and detach after 24 hr of F806-treatment, however, visible inhibition of growth was not observed until 48 hr after treatment. After 48 hr and 72 hr of treatment, the adherent cells significantly decreased, while the floating dead cells increased (Supplementary Figure 2). These results clued that F806 inhibited cell adhesion, resulting in the induction of apoptosis in a process named ‘anoikis’ [24]. When EC109 and KYSE510 cells were cultured in poly-HEMA plates in presence of F806, a significant induction of anoikis in a dose-dependent manner was found (Figure 3A). Additionally, the effect of F806 on cell adhesion was investigated in different ECM conditions and different treatments. As shown in Figure 3B, F806 significantly inhibited cell adhesion, while there was no selectivity on fibronectin (FN), collagen (COL) or laminin (LAM). Furthermore, when EC109 and KYSE510 cells were kept in FN or COL-coated plates for 30 min and treated with F806 for another 24 h (Figure 3C), or kept for 24 h prior to 30 min of F806 stimulation (Figure 3D), cell adhesion was still significantly inhibited. These data indicated that F806 clearly inhibited cell adhesion, indicating anoikis.

### F806 inhibits focal adhesion formation in ESCC cells

**Figure 4:**
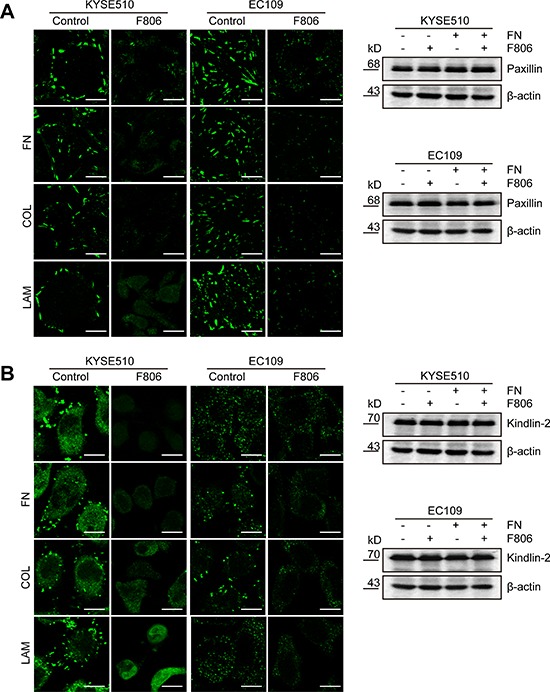
F806 inhibits focal adhesion formation in ESCC cells EC109 and KYSE510 cells were kept in FN, COL or LAM-coated coverslips in the presence of F806 for 24 h. **A.** Cells on coverslips were analyzed by immunofluorescent staining for focal adhesion marker Paxillin (*left panel*). Whole-cell lysates were immunoblotted for anti-Paxillin and β-actin (*right panel*). **B.** Cells on coverslips were analyzed by immunofluorescent staining for another focal adhesion marker Kindlin-2 (*left panel*). Whole-cell lysates were immunoblotted for anti-Kindlin-2 and β-actin (*right panel*). Original magnification, 1200×; Scale bar, 20 μm.

The results of anoikis led us to the hypothesis that adhesion complex formation might be inhibited in ESCC cells by F806. To investigate the effect of F806 on adhesion complex formation, focal adhesion marker Paxillin and Kindlin-2 was detected F806-treated cells by immunofluorescence staining. After cells on FN, COL or LAM-coated coverslips treated with F806 for 24 hr, focal adhesion were clearly observed in the basal layer of cells and the cell shapes remained normal in the control group, however, focal adhesion formation were obviously inhibited in F806-treated group (Figure 4A and 4B, left panel). The observations were supported by experiments on both of focal adhesion marker Paxillin and Kindlin-2. Additionally, the effect of F806 on expression of Paxillin and Kindlin-2 were evaluated by Western blot. As shown in Figure 4A and 4B, right panel, compared to the control, no difference on expression of Paxillin and Kindlin-2 were found in F806-treated cells. Therefore, F806 inhibited focal adhesion formation of ESCC cells and consequently inhibited cells to attach to ECM.

### F806 blocks the activation of β1 integrin in ESCC cells

**Figure 5:**
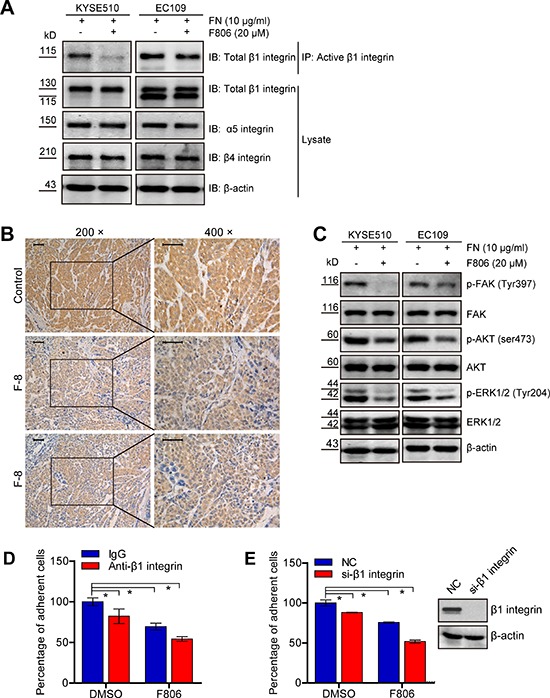
F806 inhibits β1 integrin activation in ESCC cells **A.** Cell lysates were immunoprecipitated (IP) with anti-active β1 integrin antibody followed by probing with anti-total β1 integrin antibody. Total cell lysates were immunoblotted for anti-total β1 integrin, α5-integrin, β4-integrin and β-actin. **B.** Paraffin-embedded tumor tissues from xenograft models were immunohistochemistry staining for anti-active β1 integrin. Original magnification, 400×; Scale bar, 50 μm. **C.** Total cell lysates were immunoblotted for anti-p-FAK, FAK, p-AKT, AKT, p-ERK, ERK and β-actin. **D.** KYSE 510 cells were pre-incubated with anti-β1 integrin or control IgG for 30 min and then seeded into FN-coated plates in the presence of F806 for 24 hr. The attached cells were recorded by MTT assay in each group. **E.** After si-NC or si-β1 integrin transfection for 48 hr, KYSE510 cells were kept in FN-coated plates in the presence of F806 for 24 hr. The attached cells were recorded by MTT assay in each group. *P < 0.05 *vs*. control group; mean ± SD, *n* = 3–6.

We next investigated the potential mechanism of F806 involved in the inhibition of cell adhesion. Previous studies have shown that integrins mediate cells to ECM and promote the transmission of multiple signaling pathways for the initiation of cell proliferation, including ERK/MAPK and PI3K/AKT [25, 26]. Western blot analysis revealed that no apparent effect on the total protein level of β1 integrin, β4 integrin and α5 integrin was observed; nevertheless, F806 inhibited β1 integrin activation in KYSE510 and EC109 cells by immunoprecipitation (Figure 5A). Moreover, tumor sections from xenograft models were immunochemistry stained for active β1 integrin. Compared to control group, tumors from the F806-treated group showed obvious inhibition of active β1 integrin (Figure 5B). To provide further evidence, we examined signal molecules known to be activated in the downstream of integrin signaling. Decreases in levels of activated FAK (p-FAK), AKT (p-AKT) and ERK1/2 (p-ERK1/2) were observed in F806-treated ESCC cells (Figure 5C).

To determine whether F806 inhibited the activation of β1 integrin, resulting in inhibition of cell adhesion and inducing anoikis, the blockade of the activation of β1 integrin using specific antibody JB1A and down-regulation of β1 integrin by siRNA were examined to mimic the effect of F806. As shown in Figure 5D, the blockade of the activation of β1 integrin inhibited cell adhesion in KYSE510 cells; however, F806 was more effective and the combination of anti-β1 integrin with F806 caused the most suppression of cell adhesion. Furthermore, knockdown of β1 integrin by siRNA certainly decreased the number of cell adhesion; nevertheless, F806 still was more effective. Importantly, after the knockdown of β1 integrin, mounting inhibition ratio of cell adhesion was found in F806-treated KYSE510 cells (Figure 5E). Collectively, it can be concluded that F806 would inhibit the activation of β1 integrin, leading to inhibition of focal adhesion formation and cell adhesion, finally triggering apoptosis.

## DISCUSSION

**Figure 6:**
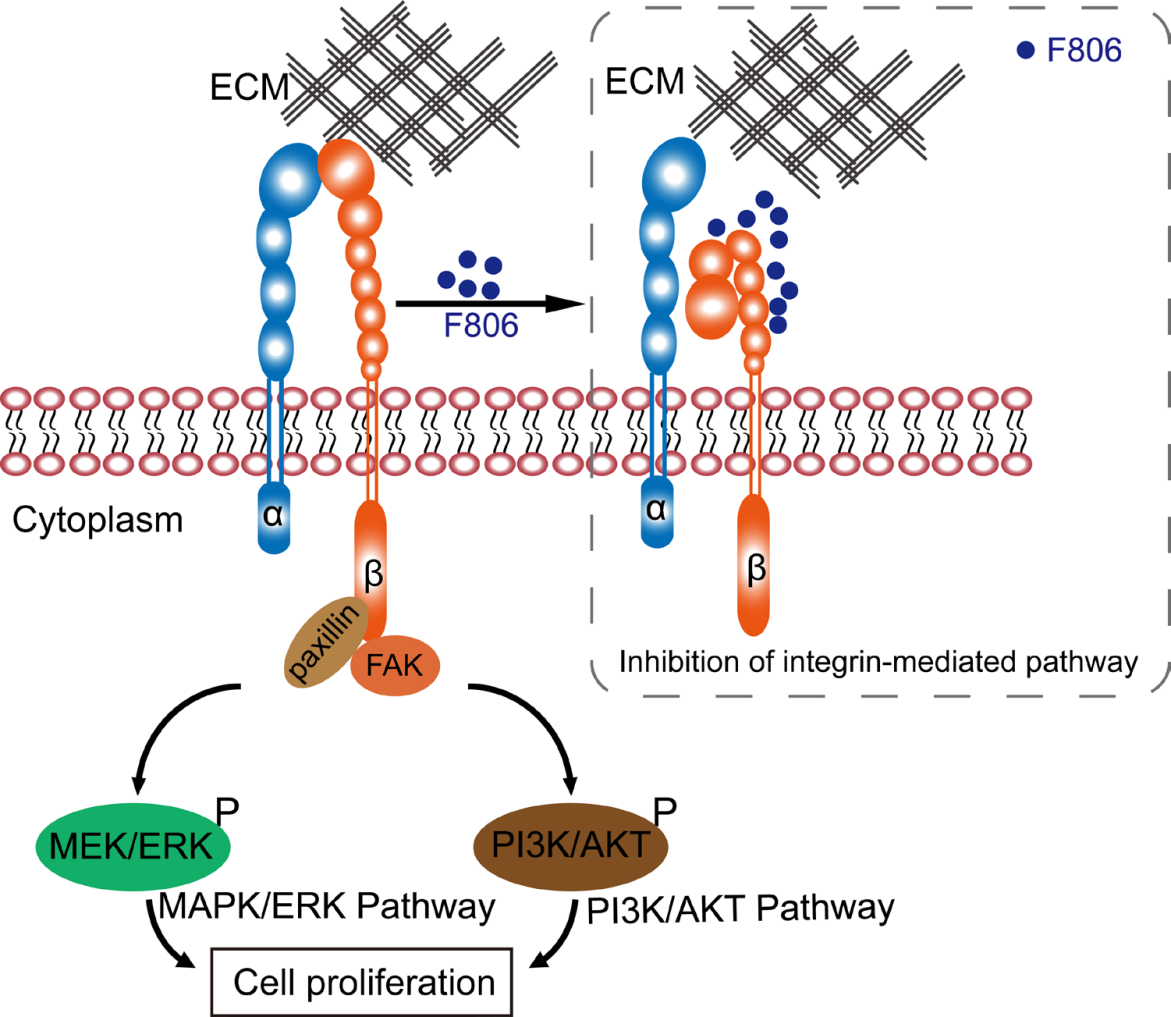
Schematic graph of the anti-cancer mechanism of F806

This study reported on the efficacy and mechanism of action of a novel macrolide analog F806 in preclinical models of esophageal squamous cell carcinoma (ESCC). Anticancer activity of F806 was evaluated in two human ESCC xenograft models and in six ESCC cell lines. Our data showed principle mechanisms for how F806 inhibited the growth in ESCC cells: F806 decreased the activation of β1 integrin, prevented cancer cells away from the ECM, and eventually initiated apoptosis (Figure 6).

In 1979, Westley JW et al. reported the isolation of the novel compound conglobatin from fermentation cultures of *Streptomyces conglobatus* ATCC 31005, which has the same molecular formula as F806; however, antifungal, antibacterial, antiprotozoal, or antitumor activity remained unclear [27]. F806, also named FW-04-806, was extracted from the China-native *Streptomyces* FIM-04-806 and demonstrated potent anticancer activity in K562 human chronic myelocytic leukemia cells, SKBR3 and MCF-7 human breast cancer cells [20–22]. In the present study, F806 displays promising antitumor activity in ESCC cells and showed very low toxicity to the non-cancerous cells. Notably, F806 reduced the volume of ESCC xenografts at a 4 mg/kg/d dose by intraperitoneal administration, which is far below the reported minimal dose of 50 mg/kg [22]. The lower dose in the treatment of ESCC models representative of lower toxicity, is a prerequisite for a successful pharmacological agent against cancer. Indeed, F806 exhibited good safety during the treatment in ESCC xenografts: no abnormality in body weight, daily diet, liver and renal function, hematological indices, and histological characteristics in the lungs, brain, heart, kidney and liver tissue, were observed (Figure 1C, Supplementary Tables 3 and 4). Considering that the *in vivo* experiments was performed only with xenograft models in this study, the efficacy and safety of F806 should be further investigated in spontaneous human cancer models.

Our previous study reported that F806, could bind to the N-terminal of Hsp90 and inhibit Hsp90/Cdc37 interaction, resulting in the disassociation of Hsp90/Cdc37/client complexes and the degradation of Hsp90 client proteins in breast cancer cells [22]. Here our observations suggested that another novel anti-cancer mechanism of F806 would exist in ESCC cells. Firstly, cell growth of ESCC cells were inhibited owing to the induction of apoptosis, which was supported by multiple evidences including the DNA laddering, the condensation and margination of nuclear chromatin surrounding through transmission electron microscope. However, cells started floating after 24 hr of treatment, while the inhibition of growth was not visibly observed until 48 hr after the treatment. This phenomenon provided clues for the hypothesis of detachment as a cause of growth inhibition and apoptosis in F806-treated ESCC cells. Indeed, anoikis induction contributed to the potent antitumor effects of F806, as evidenced by inhibition of cell adhesion to the ECM components including FN, COL and LAM. Furthermore, the inhibition of focal adhesion formation was detected by immunofluorescence, which confirmed the results. These results strongly supported the notion that F806 attenuated cell adhesion, contributing to the inhibition of cell growth, i.e., anoikis.

Upon adhesion to ECMs, integrins as a large family of heterodimeric cell surface receptors, govern cell-ECM interactions and mediate cell adhesion and provide critical signaling that regulates cellular proliferation and survival [25, 26, 28]. Previous observations proposed that chemopreventive drugs or small molecules would induce apoptosis by modulating integrin-mediated signaling [29, 30]. In our present study, no apparent effect on the protein level of β1 integrin, β4 integrin and α5 integrin was observed; nevertheless, the activation of β1 integrin was inhibited by F806 both *in vitro* and *in vivo*. Activation of integrins is essential for cell adhesion and matrix assembly [11]. An increase in the proportion of heterodimers adopting high-affinity comformation (the affinity of integrins for their ligands) was termed integrin activation [31]. It has been widely accepted that the binding of Talins and Kindlins to β1 integrin is critical for integrin activation [32–33]. Therefore, the inactivation of β1 integrin would in part inhibit the affinity of integrins for their ligands, resulting in anoikis. Classically, β1 integrin acts largely as mechanoreceptors, not only facilitating growth factor receptor signaling, but also transmitting biochemical cues that mediate multiple aspects of malignant cell behavior [34, 35]. These roles of β1 integrin prompt us to identify it as a potential target of F806 in ESCC cells. The decrease in the downstream signal molecules (such as p-FAK, p-AKT and p-ERK1/2) of integrin signaling confirmed the suppression of β1 integrin activation by F806 in ESCC cells.

The specific blockade of β1 integrin antibody was performed to determine whether the inhibition of β1 integrin activation by F806 resulted in suppression of cell adhesion and inducing anoikis. It was showed that F806 revealed greater inhibitory effect on cell adhesion in ESCC cells, in contrast to specific anti-β1 integrin JB1A. Nevertheless, the combination of F806 with JB1A could cause the most suppression of cell adhesion in ESCC cells (Figure 5D). The potential synergistic effect of F806 and JB1A on the inhibition of cell adhesion hinted different binding site between F806 and JB1A. Anti-β1 integrin JB1A was recently mapped to recognize and bind residues 82–87 of β1 integrin chain, leading to the inhibition of cell adhesion [36]. Based on these observations, docking computation model was performed to predict possible F806-binding site of β1 integrin. Indeed, it was presented that F806 would interact with Arg610 site of β1 integrin by pi bond (π bond) (Supplementary Figure 3). However, detailed investigation might be necessary to clarify the mechanism of the interaction between F806 and Arg610 site of β1 integrin.

Our study also showed that the knockdown of β1 integrin indeed decreased cell adhesion in KYSE510 cells, while F806 still was more effective than si-β1 integrin. Intriguingly, after the remarkable knockdown of β1 integrin, mounting inhibition ratio of cell adhesion was found in F806-treated KYSE510 cells (Figure 5E). Obviously, after the loss of the target molecule β1 integrin by si-RNA, F806 still could suppress KYSE510 cells binding to ECM. It hinted that β1 integrin would not be the only potential target of F806 in ESCC cells. Among the 24 different integrins, there are eight β-subunits [11]. Furthermore, other integrins has been highly attractive target in oncology therapy, such as that cilengitide is an RGD pentapeptide inhibitor of αV integrin against glioblastoma [37]. Therefore, F806 might also direct or indirect against other integrin subunit and further investigations would be warranted to verify the detailed mechanism.

Mounting experimental evidence supports that drug resistance of a targeted therapeutic agent can be acquired, because inhibition of one key pathway in a tumor may not completely turn off other parallel signaling pathways, allowing some cancer cells to survive and propagate. On this basis, Hanahan and Weinberg proposed that it could be necessary to target as many key pathways as possible, to prevent the development of adaptive resistance [38]. Potent angiogenesis inhibitors such as bevacizumab, has succeeded in suppressing tumor growth in clinical or preclinical models. However, acquired resistance can develop, resulting in increased invasion and local metastasis [39–43]. Integrin β1 signaling mediates therapeutic resistance in multiple solid cancer models [12–14]. Furthermore, targeting β1 integrin inhibits tumor growth in bevacizumab-resistant glioblastoma [13]. In this study, it was clearly demonstrated that F806 could induce apoptosis and inhibit cell growth via blocking the activation of β1 integrin. It implies that F806 could overcome resistance to antiangiogenesis therapy and would be of potential use in combination with angiogenesis inhibitors.

In summary, our data indicate that the novel drug F806 revealed promising antitumor activity in ESCC models and showed very low toxicity to the non-cancerous cells. In addition, this study highlights the inhibition of β1 integrin activation as a potential mechanism of F806, contributing to the suppression of focal adhesion formation, the prevention of further tumor growth and survival from the ECM, and the eventual initiation of apoptosis (i.e., anoikis) in ESCC cells. Therefore, F806 merits further evaluation and precise delineation as a therapeutic agent against human ESCC.

## MATERIALS AND METHODS

### Chemicals and cell lines

The compound F806, also named FW-04-806 (purity ≥ 98.5%), was produced by Fujian Institute of Microbiology, China (Supplementary Figure 1) [20, 21]. It was dissolved in DMSO at 100 mM as a stock solution and further diluted in medium before each use. The characteristics of the seven squamous cell carcinoma cell lines prepared for this study and general information are shown in Supplementary Table 1. All cells were incubated at 37°C in a humidified atmosphere containing 5% CO_2_.

### Efficacy and safety of F806 in xenograft models

Five-week-old male Nude/Nude mice were purchased from Vital River Laboratories (Beijing, China) and maintained on a 12 hr light/dark cycle under specific pathogen-free conditions, with free access to food and water. All animal studies were conducted in accordance with protocols approved by the Animal Research Committee of the Shantou Administration Center. Mice were inoculated subcutaneously with 1.0 × 10^6^ EC109 or 1.0 × 10^6^ KYSE510 human esophageal squamous cell carcinoma (ESCC) cells on the right flank. The next day, mice were randomized to three groups of 10 mice per group. Tumor growth was monitored daily, and tumor size was measured at (length × width^2^) × 0.5 twice per week. At approximately day 12 post tumor cell injection, when the tumor volume reached approximately 0.5 mm in diameter, drug administration was initiated. Mice with a tumor volume of < 0.1 mm or > 1.0 mm in diameter were excluded from drug treatment. Briefly, a 100 mg/ml F806 stock solution was prepared in 100% ethyl alcohol. On the day of injection, F806 was diluted in 0.9% NaCl containing 5% Tween-80 and 5% polyethylene glycol-400 (Sigma, St Louis MO, USA) to a final concentration such that a dose of 4 mg/kg or 8 mg/kg, in 200 μL solution, was administered to each mouse. F806 solution was intraperitoneally administered daily for 21 days. Mice were euthanized on day 22 and blood was collected for biochemical analysis or complete blood count (including differential and platelet counts). Tumors were excised, weighed, and preserved in formalin for histological analysis. The lungs, liver, heart, brains and kidneys were harvested and evaluated histologically.

### Cell viability assay

Cells inoculated in 96-well plates were treated with different concentrations of F806 for 72 hr. Changes in cell morphology were documented by photography every 24 hr. Cell viability was measured using a 3-(4, 5-dimethylthiazol-2-yl)-2, 5-diphenyltetrazolium bromide (MTT) proliferation kit (Sigma, St. Louis, USA) at 72 hr, as described by the manufacturer. The formed crystals were dissolved in dimethylsulfoxide and optical density (OD) in each well was measured at a method of dual-wavelength of 490 nm using a microplate reader. Inhibition of growth was expressed as an inhibition ratio (%) = (1 − OD490_Treated_/OD490_Control_) × 100 or a growth ratio (%) = OD490_Treated_/OD490_Control_ × 100. The half maximal inhibitory concentration (IC_50_) was calculated from the inhibition ratio and F806 concentration by SPSS 13.0 software.

### Observation by transmission electron microscopy

Cells treated with F806 for 24 hr were pre-fixed in 2% cacodylate-buffered glutaraldehyde, post-fixed in 1% osmium tetroxide, dehydrated in a graded series of alcohol, and embedded in epon. Sections were stained with uranyl acetate and lead citrate, and were examined with a JEM-1400 transmission electron microscope (JEOL, Japan).

### Dna ladder assay

Cells treated with F806 for 24 hr were harvested and the DNA was extracted with the Apoptotic DNA Ladder Kit (Beyotime Institute of Biotechnology, Shanghai, China) according to the manufacturer's protocol. DNA was analyzed by 1% agarose gel electrophoresis and visualized under UV light.

### Cell cycle analysis

Cells treated with F806 for 24 hr were harvested and were fixed with 70% cold ethanol in PBS at 4°C overnight. Subsequently, the samples were permeabilized in phosphate-buffered saline (PBS) with 0.1% Triton X-100 and stained again with a solution containing 5 μg /mL propidium iodide (PI) and 50 μg/mL RNase, for 30 min at room temperature in the dark. Finally, the samples were immediately analyzed by flow cytometry using a Coulter Epics XL flow cytometer (Beckman-Coulter Inc, Brea, CA, USA). Fluorescence emitted from the PI-DNA complex was determined by FlowJo software (TreeStar, San Carlos, CA, USA). Cell cycle distribution was calculated using Multicycle software (Phoenix Flow Systems, San Diego, CA, USA).

### Western blot analysis

After cells were treated with F806 for 24 hr, total cell lysates were prepared in Laemmli sample buffer (Bio-Rad, 101-0737, Hercules, CA, USA). Western blots were performed as described previously [44]. Primary antibodies to ERK1/2, α5 integrin, β4 integrin and β-actin were purchased from Santa Cruz Biotechnology (Santa Cruz, CA, USA), while antibodies to PARP, phospho-ERK1/2 (p-ERK1/2, Tyr204), phospho-AKT (p-AKT, Ser473) and AKT were purchased from Cell Signaling Technology (Danvers, MA, USA), along with phospho-FAK (p-FAK, Tyr397) from Invitrogen (Camarillo, CA, USA), Kindlin-2 from OriGene Technologies (Rockville, MD, USA), FAK, paxillin, and β1 integrin from BD Transduction Laboratories (Franklin Lakes, New Jersey, USA). Appropriate HRP-conjugated secondary antibodies were from Santa Cruz Biotechnology (Dallas, Texas, USA). Detailed information about the antibodies is listed in Supplementary Table 2.

### Terminal deoxynucleotidyl transferase-mediated dUTP nick end labeling (TUNEL) assay

The DeadEnd™ Fluorometric TUNEL System (Promega, Madison, USA) was used to detect apoptosis in paraffin-embedded tumor tissues following the manufacturer's protocol. These tissue sections were subsequently stained for nuclei with 500 ng/mL propidium iodide (PI). The samples were immediately analyzed under a fluorescence microscope using a standard fluorescent filter set to view the green fluorescence at 520 nm and red fluorescence of PI at > 620 nm. The slides were imaged using an Openlab controlled microscope.

### Anoikis assay

For determination of anoikis, 2 × 10^5^ cells were cultured on 24-well plates coated with poly (2-hydroxyethyl methacrylate) (poly-HEMA) (Cell Biolabs, Inc., CBA-080, San Diego, CA, USA) in presence of various F806 for 72 hr. Cell viability was determined by MTT colorimetric detection and presented as mean percentage of control sample plus or minus standard deviation (SD). All experiments were performed in triplicate.

### Adhesion assay

Ninety-six well plates were coated with Fibronectin (10 μg/mL, Sigma, F-2006, St. Louis, MO, USA), Collagen IV (10 μg/mL, Sigma, C-7521) or Laminin (10 μg/mL, Sigma, L-2020) at 4°C overnight. After removal of excess coating buffer and one wash with PBS, nonspecific binding sites were blocked for 1 hr in 0.1% bovine serum albumin (BSA) in phosphate-buffered saline (PBS) at room temperature, following with a second wash with PBS. EC109 and KYSE510 cells (2 × 10^4^) in 100 μL serum-free medium with or without F806 per well, were allowed to adhere for 24 hr at 37 C in a humidified 5% CO2 incubator. In the adhesion inhibition assay, antibody JB1A against the β1 integrin (1:100, Millipore, MAB1965, Bedford, MA) blocking cell adhesion to Fibronectin, was added to KYSE510 cells for 30 min prior to seeding. Nonspecific adherent cells were removed by washing with PBS three times. Adherent cells were subsequently measured by MTT colorimetric detection. Cell adhesion was presented as mean percentage of control sample plus or minus standard deviation (SD). All experiments were performed in triplicate.

### Immunofluorescence

Cells grown on coverslips coated or uncoated with 10 μg/mL fibronectin, collagen IV or Laminin at 4°C overnight, were treated with F806 for 24 hr. After incubation, cells were fixed with 4% paraformaldehyde in PBS for 20 min, and permeabilized with 0.1% Triton X-100 in PBS for 10 min. After blocking with 5% donkey serum in PBS for 60 min, cells were stained with mouse anti-paxillin or mouse anti-kindlin-2 at 4°C overnight. After washing, samples were incubated with Alexa Fluor 488-AffiniPure Donkey anti-mouse IgG (Jackson, 711-545-150, Newmarket, Suffolk, UK, 1:200). Cells were analyzed using an Olympus FV1000 confocal microscope (Olympus, Tokyo, Japan).

### Measurement of the activity of β1 integrin by immunoprecipitation

Measurement of β1 integrin activity by immunoprecipitation was performed as described previously [45, 46]. Cells were inoculated on fibronectin-coated (10 μg/mL) cell culture dishes, in the absence or presence of 20 μM F806 for 24 hr. Cells were lysed in 4°C Triton lysis buffer (20 mM Tris-HCl, pH 7.4, 150 mM NaCl, 1% Triton X-100, and 5 mM EDTA) with 1X Halt Protease and Phosphatase Inhibitor Cocktail (Thermo Fisher Scientific Inc., 78441, Rockford, IL, USA). Cell lysates were immunoprecipitated with anti-active β1 integrin (CHEMICON International, Inc., MAB2079Z, Billerica, MA, USA), rotating overnight at 4°C. Immune complexes were sedimented (3000 rpm for 3 minute at 4°C) and washed five times with ice-cold lysis buffer. The pelleted immune complexes were resuspended in 1× Laemmli sample buffer and boiled before being resolved by SDS-PAGE.

### Statistical analyses

Data analysis was performed using SPSS 13.0 software (SPSS, Inc., Chicago, IL, USA). A two-way ANOVA was used to determine the significance of differences between groups and *P* < 0.05 was deemed statistically significant. Data were plotted as mean ± SD at least three independent experiments using GraphPad Prism 5 software (GraphPad Software, Inc., La Jolla, CA, USA).

## SUPPLEMENTARY FIGURES AND TABLES


