# Supplementary material for: Macrolide analog F806 suppresses esophageal squamous cell carcinoma (ESCC) by blocking β1 integrin activation
Source: Oncotarget. 2015 Apr 3;6(18):15940–52. doi: 10.18632/oncotarget.3612 (PMC4599248; doi:10.18632/oncotarget.3612)
Supplement: Supplementary file 1 [file oncotarget-06-15940-s001.pdf]

SUPPLEMENTARY FIGURES AND TABLES

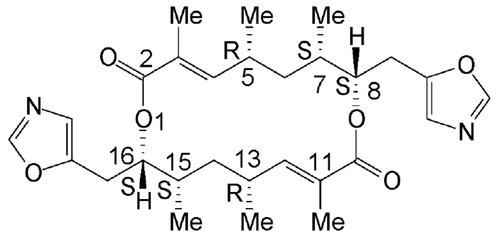

Supplementary Figure S1: The chemical structure diagram of F806. The formula of F806 is $C_{28}H_{38}N_2O_6$.

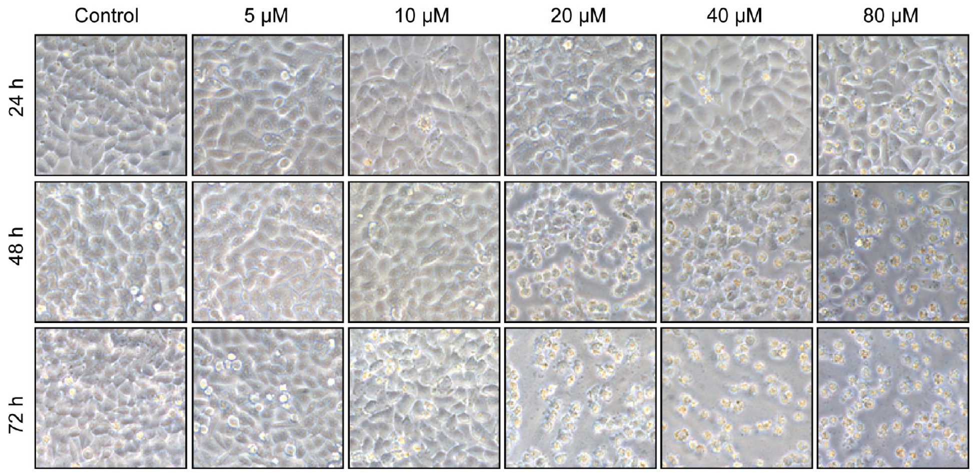

Supplementary Figure S2: Representative morphological features of F806-treated EC109 cells (Original magnification, 200×). Various ESCC cells were treated with 0–80 μM F806 for 72 hours and changes in cell morphology were documented by photography every 24 hr.

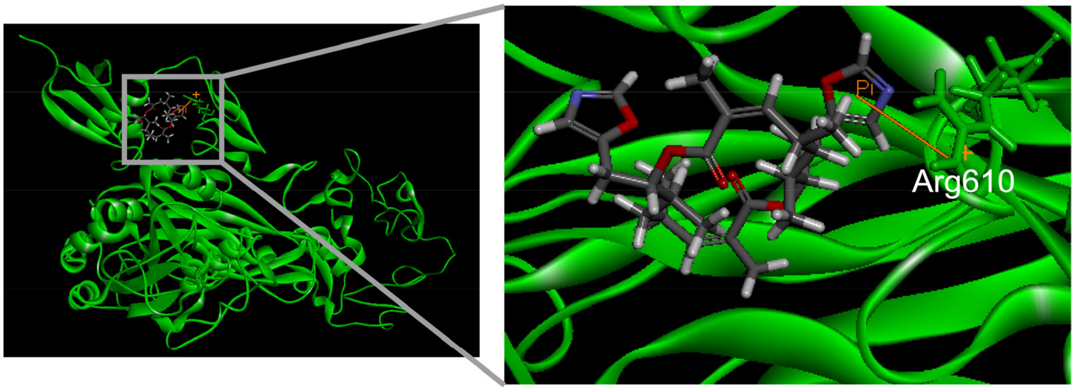

Supplementary Figure S3: Molecular modeling of surface area diagram indicating Arg610 site of β1 integrin potentially involved in direct interaction with F806. The 3D structure model of F806 was obtained from PubChem Compound of National Center for Biotechnology Information (NCBI) (Conglobatin, CID_6440452). The homology structure model of β1-integrin was generated with SWISS-MODEL based on primary amino acid sequence of integrin beta-1 isoform 1A precursor from GenBank of NCBI (protein ID: NP_002202.2). The molecular docking study of F806 with β1-integrin was performed using Discovery Studio 2.5. Default variables were used according to the manual of Discovery Studio.

Supplementary Table S1. Summary of cell lines and cell culture general information in this study

Cell line Name	Description	*Culture medium	Cell source	Ref.
EC109	Esophageal squamous cell carcinoma cells	[1]DMEM with 10% [2]NBS	purchased from Type Culture Collection of Chinese Academy of Sciences	(1)
KYSE70	Esophageal squamous cell carcinoma cells	[3]1640 with 10% [4]FBS	gift from Professor Ming-Zhou Guo	(2)
KYSE450	Esophageal squamous cell carcinoma cells			
KYSE150	Esophageal squamous cell carcinoma cells			
KYSE180	Esophageal squamous cell carcinoma cells			
KYSE510	Esophageal squamous cell carcinoma cells			
MTLn3	Rat mammary adenocarcinoma cells	DMEM /[5] F12 with 10% NBS	gift from Professor Wei Gu (Shantou University)	(3)

*Culture medium, various media were supplemented with penicillin-G (100 units/mL) and streptomycin (100 µg/mL); [1]DMEM, Dulbecco's modified Eagle's medium (GIBCO); [2]NBS, Newborn Bovine Serum (Excell biology. Inc. Shanghai, China); [3]1640, 1640 medium (Thermo, Waltham, MA, USA); [4]FBS, Fetal Bovine Serum (Thermo, Waltham, MA, USA); [5]F12, F-12 nutrient mixture (Ham) powder (GIBCO); Ref., Reference.

REFERENCES

1. Pan QQ. Studies on esophageal cancer cells in vitro. Proc. Chin. Acad. Med. Sci. Peking Union Med Coll. 1989; 4:52–7.
2. Shimada Y, Imamura M, Wagata T, Yamaguchi N, Tobe T. Characterization of 21 newly established esophageal cancer cell lines. Cancer. 1992; 69:277–84.
3. Neri A, Welch D, Kawaguchi T, Nicolson GL. Development and biologic properties of malignant cell sublines and clones of a spontaneously metastasizing rat mammary adenocarcinoma. Journal of the National Cancer Institute. 1982; 68:507–17.

Supplementary Table S2. Detailed information about the antibodies used in this study

Antibody to	Mono/polyclonal	Company (catalogue number)	Application(dilution)
PARP	Rabbit monoclonal	Cell Signaling Technology (9542)	Western blot (1:1000)
β-actin	Mouse monoclonal	Santa Cruz Biotechnology (sc-47778)	Western blot (1:2000)
p-AKT(Ser473)	Rabbit monoclonal	Cell Signaling Technology (4060)	Western blot (1:2000)
AKT	Rabbit monoclonal	Cell Signaling Technology (4691)	Western blot (1:2000)
p-ERK1/2 (Tyr204)	Mouse monoclonal	Santa Cruz Biotechnology (sc-7383)	Western blot (1:1000)
ERK1/2	Rabbit polyclonal	Santa Cruz Biotechnology (sc-94)	Western blot (1:2000)
p-FAK (Tyr397)	Rabbit monoclonal	Invitrogen (44-625G)	Western blot (1:1000)
FAK	Mouse monoclonal	BD Transduction Laboratories (610088)	Western blot (1:1000)
β1 integrin	Mouse monoclonal	BD Transduction Laboratories (610468)	Western blot (1:1000)
Paxillin	Mouse monoclonal	BD Transduction Laboratories (610620)	Western blot (1:2000)<br>Immunofluorescence (1:200)
Kindlin-2	Mouse monoclonal	OriGene Technologies, Inc. (TA500505)	Western blot (1:2000)<br>Immunofluorescence (1:200)
Active β1 integrin	Mouse monoclonal	CHEMICON International, Inc. (MAB2079Z)	IP (2 μl in each 500 μg protein)
α5 integrin	Rabbit polyclonal	Santa Cruz Biotechnology (sc-10729)	Western blot (1:1000)
β4 integrin	Rabbit polyclonal	Santa Cruz Biotechnology (sc-9090)	Western blot (1:1000)
Anti-mouse IgG-HRP	goat	Santa Cruz Biotechnology (sc-2005)	Western blotting (1:7500)
Anti-rabbit IgG-HRP	goat	Santa Cruz Biotechnology (sc-2030)	Western blotting (1:7500)

Supplementary Table S3. Serum analysis for liver and renal function (mean ± SD, *n* = 6)

Group	AST (U/L)	ALT (U/L)	TBil (μmol/L)	ALB (g/L)	BUN (mmol/L)	Cr (μmol/L)
Control	205.3±52.8	41.8±7.7	8.4±4.6	18.2±1.0	5.6±0.9	21.5±4.1
F-4	205.3±29.0	39.7±8.2	11.8±4.9	19.5±1.0	5.9±0.9	19.8±3.5
F-8	141.7±16.6	34.3±23.7	9.9±3.3	19.2±1.8	5.6±0.5	18.1±2.5

Blood samples were collected via the ocular vein. Then serum was obtained by centrifugation of whole blood at 3000 rpm for 15 min. Liver function was evaluated based on the serum levels of aspartate aminotransferase (AST), alanine aminotransferase (ALT), total bilirubin (TBil) and albumin (ALB). Nephrotoxicity was determined by blood urea nitrogen (BUN) and creatinine (Cr). All biochemical parameters were determined by an automated biochemical analyzer (Beckman).

Supplementary Table S4. Routine analysis of complete blood count with differential (mean ± SD, *n* = 5)

Group	Control	F-4	F-8
WBC (×10^9/L)	2.9 ± 1.5	2.8 ± 1.1	3.3 ± 0.9
RBC(×10^{12}/L)	9.2 ± 0.4	8.9 ± 0.3	8.7 ± 0.3
HGB (g/L)	149.4 ± 6.3	146.8 ± 6.4	145.6 ± 4.7
HCT	0.5 ± 0.0	0.5 ± 0.0	0.4 ± 0.0
MCV (fL)	54.3 ± 1.8	51.4 ± 2.2	50.6 ± 2.2
MCH (pg)	16.5 ± 0.8	16.6 ± 0.8	16.7 ± 0.3
MCHC (g/L)	303.9 ± 9.6	322.0 ± 3.4	331.4 ± 10.2
PLT (×10^9/L)	902.4 ± 141.3	825.0 ± 176.0	788.3 ± 217.0

Blood samples were collected via the ocular vein. Complete blood count (including differential and platelet counts) was determined by an automated blood cell analyzer (Beckman).WBC, White blood count; RBC, Red blood count; HGB, hemoglobin; HCT, hematocrit; MCV, Mean corpuscular volume; MCH, Mean corpuscular hemoglobin; MCHC, Mean corpuscular haemoglobin concentration; PLT, Blood platelet count.
